# Supplementary material for: Modeling glioblastoma heterogeneity as a dynamic network of cell states
Source: Mol Syst Biol. 2021 Sep 16;17(9):e10105. doi: 10.15252/msb.202010105 (PMC8444284; doi:10.15252/msb.202010105)
Supplement: Supplementary file 6 — Source Data for Figure 5 [file MSB-17-e10105-s004.zip › Figure5A_sourcedata/GSEA_3071/hallmarks_stateIII.GseaPreranked.1623742540963/HALLMARK_MYOGENESIS.html]

Details for gene set HALLMARK\_MYOGENESIS[GSEA]

|  || Dataset | state33071 |
| Phenotype | NoPhenotypeAvailable |
| Upregulated in class | na\_neg |
| GeneSet | HALLMARK\_MYOGENESIS |
| Enrichment Score (ES) | -0.4936664 |
| Normalized Enrichment Score (NES) | -2.7325535 |
| Nominal p-value | 0.0 |
| FDR q-value | 0.0 |
| FWER p-Value | 0.0 |
Table: GSEA Results Summary

  

Fig 1: Enrichment plot: HALLMARK\_MYOGENESIS      
 Profile of the Running ES Score & Positions of GeneSet Members on the Rank Ordered List

  

| PROBE | GENE SYMBOL | GENE\_TITLE | RANK IN GENE LIST | RANK METRIC SCORE | RUNNING ES | CORE ENRICHMENT || 1 | TPM3 |  |  | 109 | 0.404 | -0.0142 | No |
| 2 | TAGLN |  |  | 407 | 0.257 | -0.0820 | No |
| 3 | HBEGF |  |  | 441 | -0.251 | -0.0818 | No |
| 4 | MYLK |  |  | 479 | -0.253 | -0.0826 | No |
| 5 | MAPK12 |  |  | 770 | -0.268 | -0.1482 | No |
| 6 | PKIA |  |  | 854 | -0.272 | -0.1602 | No |
| 7 | SCHIP1 |  |  | 995 | -0.279 | -0.1867 | No |
| 8 | PC |  |  | 1064 | -0.283 | -0.1944 | No |
| 9 | WWTR1 |  |  | 1080 | -0.284 | -0.1885 | No |
| 10 | GAA |  |  | 1151 | -0.287 | -0.1966 | No |
| 11 | DMPK |  |  | 1260 | -0.293 | -0.2143 | No |
| 12 | ADCY9 |  |  | 1286 | -0.294 | -0.2106 | No |
| 13 | PRNP |  |  | 1309 | -0.295 | -0.2061 | No |
| 14 | KCNH2 |  |  | 1384 | -0.299 | -0.2148 | No |
| 15 | PSEN2 |  |  | 1421 | -0.301 | -0.2137 | No |
| 16 | ATP6AP1 |  |  | 1471 | -0.304 | -0.2158 | No |
| 17 | CDKN1A |  |  | 1631 | -0.314 | -0.2460 | No |
| 18 | GPX3 |  |  | 1638 | -0.315 | -0.2366 | No |
| 19 | SORBS1 |  |  | 1691 | -0.318 | -0.2390 | No |
| 20 | SCD |  |  | 1737 | -0.321 | -0.2395 | No |
| 21 | AEBP1 |  |  | 1763 | -0.322 | -0.2348 | No |
| 22 | LAMA2 |  |  | 1786 | -0.324 | -0.2293 | No |
| 23 | ABLIM1 |  |  | 1940 | -0.332 | -0.2573 | No |
| 24 | NOTCH1 |  |  | 2024 | -0.337 | -0.2671 | No |
| 25 | FKBP1B |  |  | 2065 | -0.339 | -0.2657 | No |
| 26 | MAPRE3 |  |  | 2102 | -0.342 | -0.2631 | No |
| 27 | SH2B1 |  |  | 2438 | -0.368 | -0.3369 | No |
| 28 | STC2 |  |  | 2570 | -0.379 | -0.3576 | No |
| 29 | SOD3 |  |  | 2860 | -0.404 | -0.4182 | No |
| 30 | SVIL |  |  | 2912 | -0.409 | -0.4172 | No |
| 31 | BHLHE40 |  |  | 2981 | -0.417 | -0.4203 | No |
| 32 | SLC6A8 |  |  | 3004 | -0.420 | -0.4114 | No |
| 33 | NAV2 |  |  | 3180 | -0.441 | -0.4413 | No |
| 34 | ACSL1 |  |  | 3221 | -0.446 | -0.4362 | No |
| 35 | COL15A1 |  |  | 3317 | -0.464 | -0.4447 | No |
| 36 | SYNGR2 |  |  | 3356 | -0.472 | -0.4382 | No |
| 37 | CKB |  |  | 3572 | -0.536 | -0.4751 | Yes |
| 38 | ITGB1 |  |  | 3574 | -0.537 | -0.4568 | Yes |
| 39 | IGFBP7 |  |  | 3611 | -0.554 | -0.4469 | Yes |
| 40 | ERBB3 |  |  | 3617 | -0.558 | -0.4288 | Yes |
| 41 | CDH13 |  |  | 3656 | -0.583 | -0.4184 | Yes |
| 42 | COL4A2 |  |  | 3664 | -0.586 | -0.3999 | Yes |
| 43 | COL6A2 |  |  | 3674 | -0.595 | -0.3816 | Yes |
| 44 | ITGA7 |  |  | 3730 | -0.639 | -0.3737 | Yes |
| 45 | EPHB3 |  |  | 3775 | -0.706 | -0.3606 | Yes |
| 46 | APP |  |  | 3779 | -0.710 | -0.3368 | Yes |
| 47 | FST |  |  | 3782 | -0.714 | -0.3126 | Yes |
| 48 | SSPN |  |  | 3808 | -0.756 | -0.2929 | Yes |
| 49 | SPARC |  |  | 3811 | -0.759 | -0.2671 | Yes |
| 50 | NCAM1 |  |  | 3821 | -0.773 | -0.2427 | Yes |
| 51 | CLU |  |  | 3846 | -0.827 | -0.2202 | Yes |
| 52 | CHRNA1 |  |  | 3864 | -0.877 | -0.1942 | Yes |
| 53 | CRYAB |  |  | 3894 | -1.078 | -0.1644 | Yes |
| 54 | COL6A3 |  |  | 3900 | -1.171 | -0.1251 | Yes |
| 55 | COL1A1 |  |  | 3902 | -1.191 | -0.0842 | Yes |
| 56 | IGFBP3 |  |  | 3903 | -1.199 | -0.0427 | Yes |
| 57 | COL3A1 |  |  | 3918 | -1.418 | 0.0028 | Yes |
Table: GSEA details [plain text format]

  

Fig 2: HALLMARK\_MYOGENESIS: Random ES distribution      
 Gene set null distribution of ES for **HALLMARK\_MYOGENESIS**
